# Roles of the CXCL8-CXCR1/2 Axis in the Tumor Microenvironment and Immunotherapy

**DOI:** 10.3390/molecules27010137

**Published:** 2021-12-27

**Authors:** Zhi-Jian Han, Yang-Bing Li, Lu-Xi Yang, Hui-Juan Cheng, Xin Liu, Hao Chen

**Affiliations:** 1 The Key Laboratory of the Digestive System Tumors of Gansu Province, Tumor Center, Lanzhou University Second Hospital, Lanzhou 730000, China; liyangbing3906@yeah.net (Y.-B.L.); momoer1988@yeah.net (L.-X.Y.); chenghj20@lzu.edu.cn (H.-J.C.); 2 The Second Clinical Medical College, Lanzhou University, Lanzhou 730000, China; liux21@lzu.edu.cn

**Keywords:** interleukin-8, chemokine, tumor microenvironment, cancer-associated fibroblast, microbiome, CXC receptors 1 and 2, myeloid-derived suppressor cells, immunotherapy, neutrophile

## Abstract

In humans, Interleukin-8 (IL-8 or CXCL8) is a granulocytic chemokine with multiple roles within the tumor microenvironment (TME), such as recruiting immunosuppressive cells to the tumor, increasing tumor angiogenesis, and promoting epithelial-to-mesenchymal transition (EMT). All of these effects of CXCL8 on individual cell types can result in cascading alterations to the TME. The changes in the TME components such as the cancer-associated fibroblasts (CAFs), the immune cells, the extracellular matrix, the blood vessels, or the lymphatic vessels further influence tumor progression and therapeutic resistance. Emerging roles of the microbiome in tumorigenesis or tumor progression revealed the intricate interactions between inflammatory response, dysbiosis, metabolites, CXCL8, immune cells, and the TME. Studies have shown that CXCL8 directly contributes to TME remodeling, cancer plasticity, and the development of resistance to both chemotherapy and immunotherapy. Further, clinical data demonstrate that CXCL8 could be an easily measurable prognostic biomarker in patients receiving immune checkpoint inhibitors. The blockade of the CXCL8-CXCR1/2 axis alone or in combination with other immunotherapy will be a promising strategy to improve antitumor efficacy. Herein, we review recent advances focusing on identifying the mechanisms between TME components and the CXCL8-CXCR1/2 axis for novel immunotherapy strategies.

## 1. Introduction

The tumor microenvironment (TME) has a close relationship with carcinogenesis but also provides fertile ground for tumor progression and metastasis [1,2]. However, our knowledge of cellular composition, organization, spatial distribution, interaction, and communication is limited concerning the TME. A great deal of evidence has confirmed the roles of interleukins [3] or chemokines, especially the CXCL8-CXCR1/2 axis, in proinflammation, tumor enhancement, and immunotherapy resistance [4]. 

Immunotherapy is proving effective in both hematological malignant and solid tumors [5]. Some therapies [6] include adoptive cellular transfer, cancer vaccines, and immune agonists; immune checkpoint inhibitors are effective in certain patients, but most patients have innate resistance or quickly acquire resistance [7]. A novel strategy or combination therapy response in vitro shows only a modest effect in vivo [8,9]. All of these indicate a possible resistance mechanism associated with the TME and more strategies to enhance the immunotherapy are very desirable [10]. Targeting the TME [11] instead of cancer cells has proved to be a successful therapeutic method, such as with the use of antiangiogenic drugs. 

Two main research strategies relate to the TME. One has focused on the unique physicochemical characteristics of TME including hypoxia, acidic pH, enhanced vascular permeability, and abnormal nutrient partitioning [12]. The other focuses on the components of the TME, especially cellular interactions [13]. Various cells in the TME build up a complex social network. Cytokines and chemokines have an important role in the communication of these cells through the interaction to their specific receptors. Intercellular interactions in the TME play central roles in determining the molecular mechanism of disease and response to therapy [14].

Here, we review the role of the CXCL8-CXCR1/2 axis in shaping the TME and its effect in cancer therapy, especially through interaction with CAFs, the microbiome, and immune cells. Advanced therapeutic approaches are discussed, including small molecule inhibitors and antibodies targeting the CXCL8-CXCR1/2 axis.

## 2. Composition of TME

**Figure 1:**
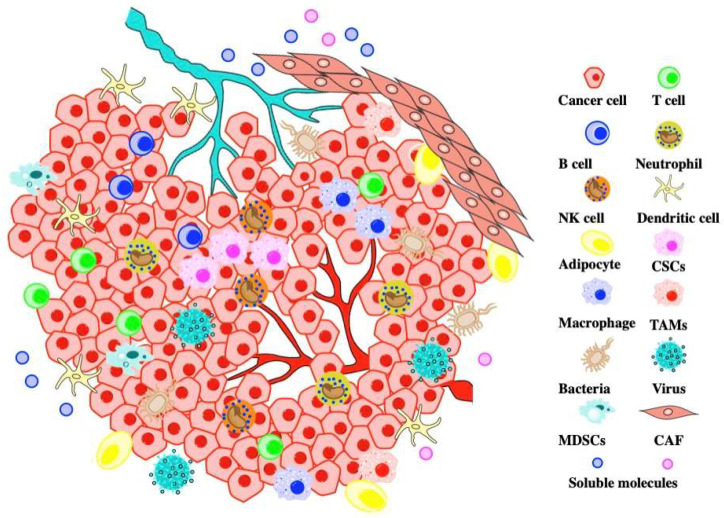
Various types of components in the TME of solid malignancies. The cellular composition of TME is mainly CAFs, cancer cells, immune cells, and the microbiome. All of the cells interact with each other via soluble molecules such as cytokines or chemokines and trafficking through blood vessels or lymphatic vessels (the abundance of each component is not illustrated here).

The major components of the TME (Figure 1) are cancer cells, stromal cells, the extracellular matrix (ECM), blood vessels, lymphatic vessels, the microbiome, and signal molecules [15]. The stromal cells are mainly cancer-associated fibroblasts (CAFs), mesenchymal stem cells (MSCs), inflammatory cells or immune cells, vascular endothelial cells, pericytes, smooth muscle cells, and adipocytes [16]. Cancer progression often benefits from the presence of CAFs, myeloid-derived suppressor cells (MDSCs), and an immune repression TME. Non-malignant CAFs can also be targeted for cancer radionuclide imaging and therapy [17]. Stromal-immune niches were temporal and spatially organized in the TME [18,19]. Immune cells are the protagonist of tumor immunotherapy, including T cells, B cells, natural killer cells (NK), dendritic cells (DCs), neutrophils, macrophages, tumor-associated macrophages (TAMs), and MDSCs. CXCL8 can recruit myeloid cells to the TME and play a key role in forming the immunosuppressive microenvironment. Ample evidence showed that MDSCs and TAMs exert their tumor-promoting effects by suppressing T-cell functions and enhancing tumor proliferation, invasion, and metastasis. All these cells and microbiomes have a close relationship with cytokines and chemokines [20]. CXCL8-CXCR1/2 can interfere with the differentiation and function of stromal cells and immune cells in TME, ultimately affecting immunotherapy [21].

## 3. CXCL8-CXCR1/2 Signaling Pathway

The proinflammatory cytokine CXCL8 was initially found as a chemotactic agent for neutrophils in inflammatory diseases. Through both autocrine and paracrine signaling, the CXCL8-CXCR1/2 axis can recruit the neutrophil to clear bacteria and protect the host from infection. Due to the similarity of the pathogenesis of inflammatory diseases and cancer, more researchers have focused on the roles of the CXCL8-CXCR1/2 axis in cancer [22]. 

**Figure 2:**
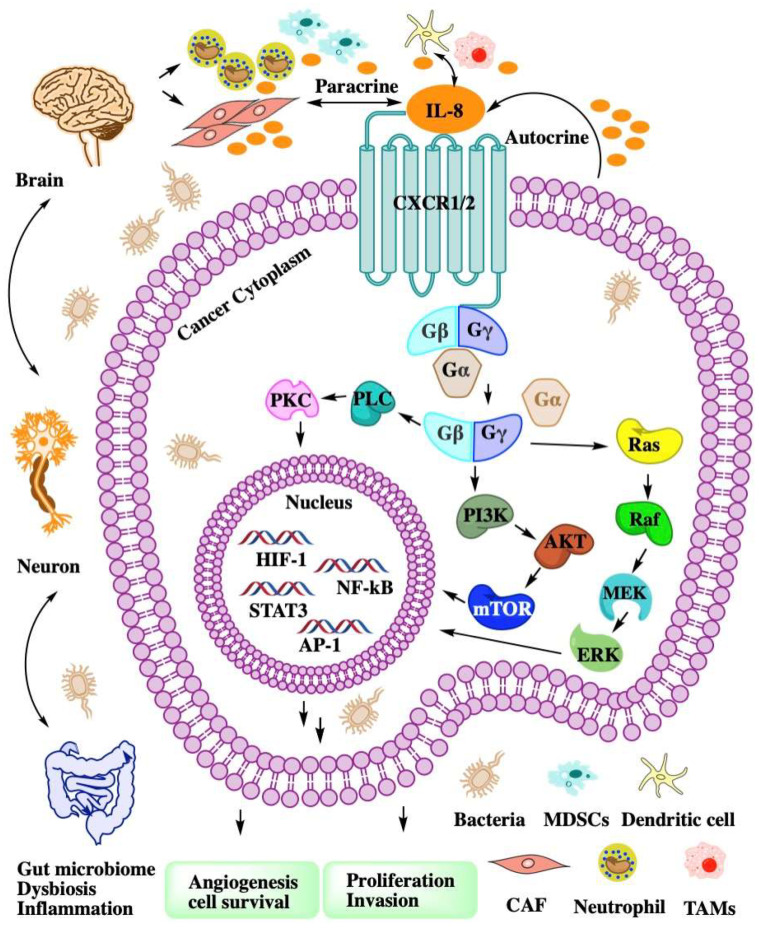
The intricate network of the CXCL8-CXCR1/2 axis in TME. CXCL8 binding to CXCR1/2 activates G-protein-mediated signaling cascades in cancer cell. CXCR1/2 activation leads to the dissociation of the Gα subunit from the Gβγ subunits. The signal of Gβγ subunits activate kinase to enhance angiogenesis, proliferation, and invasion. Cancer cell autocrine CXCL8 to recruit MDSCs or neutrophil to TME. Dysbiosis or inflammation affects myeloid cell recruitment through the gut–brain axis.

CXCL8 is a peptide with 72 amino acids and has a critical N-terminal motif of Glu-Leu-Arg (ELR). CXCL8 can be secreted by fibroblasts, CAFs, endothelial cells, epithelial cells, DCs, monocytes, macrophages, and cancer cells [23]. CXCL8 signals through two cell-surface receptors of CXCR1 and CXCR2. CXCR1 and CXCR2 are the G-protein-coupled receptor for a group of C-X-C chemokines. CXCR1 interacts with CXCL6 and CXCL8, whereas CXCR2 interacts with CXCL1, CXCL2, CXCL3, CXCL5, CXCL6, CXCL7, and CXCL8. CXCL8-CXCR1/2 signaling with CAF, the microbiome, and immune cell help in recruiting granulocytes such as neutrophils and MDSCs to the site of the TME and contribute to tumor growth by enhancing angiogenesis and promoting cancer cell proliferation [24] and immune resistance [25] (Figure 2).

### 3.1. Interactions of the CXCL8-CXCR1/2 Axis and CAF

CAFs are activated fibroblast populations and the major cellular components of the TME in primary and metastatic cancers [26,27]. CAFs have different features from normal fibroblasts after infiltrating tumor tissue, such as enhanced proliferation and epigenetic changes to produce secreted factors [28]. CAFs are contributors to desmoplasia that can facilitate tumorigenicity, cancer cell proliferation, metastasis, and cancer immunotherapy resistance through complex interactions and intricate signaling [29] with other cell types in the TME [30]. 

The population of CAFs are highly heterogeneous because several progenitor cell types can be reprogrammed into CAFs [31]. Quiescent or resident fibroblasts are major progenitor cells of CAFs [32]. Hepatic [33] or pancreatic stellate cells [34] are the putative origin of CAFs. Adipocytes [35], endothelial cells [36], epithelial cells [37], and bone marrow cells [38] can be reprogrammed into CAFs. CAFs can also derive from multiple resident precursors, such as smooth muscle cells or mesenchymal stem cells [39]. Nevertheless, the precise origins of CAFs remain elusive because of the lack of lineage biomarkers. 

CAFs have both tumor-promoting and tumor-suppressive functions [40]. The tumor-suppressive functions of CAFs [41] remain poorly understood. Part of the host defensive mechanism involves promotion of anticancer immunity, tumor-inhibitory signaling, tumor-restraining metabolism, and ECM-related physical barriers to tumor cell invasion and dissemination. Desmoplasia is the growth of fibrous or connective tissue in desmoplastic breast, lung, and pancreatic cancers. The desmoplastic reaction may form the desmoplasia or dense fibrosis around the tumor to restrict its growth and migration [42]. The mechanisms of tumor-promoting roles of CAFs are mainly regulatory functions via growth factors, cytokines, and chemokines contributing to angiogenesis; ECM remodeling; aberrant stroma, and an immunosuppressive TME [43]. Mass cytometry and single-cell analysis of pancreatic tumors and healthy pancreas samples found two stable and functionally distinct pancreatic fibroblast lineages. CD105-positive pancreatic fibroblasts promote tumor growth, but CD105-negative fibroblasts are highly tumor suppressive in a manner dependent on adaptive immunity [44].

CAF subsets have specialized secretory functions, such as cytokines, chemokines, and ECM molecules collagen I, which contribute to ECM remodeling and immunomodulatory function. Tumor–fibroblast interactions via soluble factors determine the final outcome of the tumorigenic process and affect the cancer therapy [45]. The CAF population is heterogeneous according to the cell origins, and the functional heterogeneity can be regulated by paracrine molecules such as CXCL8 and CXCR1/2 ligands [30]. CAFs can attract monocytes by secreting CXCL8 to enhance TAM enrichment and suppress NK cells’ function in colorectal cancer [46]. In gastric cancer tissues of chemoresistant patients, CXCL8 was highly expressed and located in CAFs by immunohistochemistry assay. A high serum CXCL8 level was associated with poor response to cisplatin therapy in gastric cancer patients [47]. CAFs are major sources of chemokines (CXCL1 and CXCL8) that recruit granulocytes (TAM and PMN-MDSC) to tumors. Combining a selective CSF1R inhibitor (JNJ-40346527) with a CXCR2 antagonist (SB225002) blocked granulocyte recruitment and demonstrated a strong antitumor effect, which was further improved by the addition of antiprogrammed cell death protein 1 (PD-1) [48]. 

Chronic inflammation and proinflammatory cytokines tumor necrosis factor α (TNFα) and interleukin 1β (IL-1β) can induce the conversion of MSCs to inflammatory CAFs. These CAFs secrete prometastatic chemokines including CXCL6 and CXCL8 in Luminal-A breast cancer cells and enhance migration [49]. CXCL8 can induce normal ovarian fibroblasts to CAFs and stimulate xenograft tumor growth in mice. The ovarian cancer cell stemness was promoted by the CXCL8 secreted from CAFs through the Notch3 signaling pathway [50]. Gastric cancer extracellular vesicles transfer various miRNAs and induce chemokines such as CXCL1 and CXCL8 expression in CAFs. Aberrant chemokine CXCL1 and CXCL8 expression in CAFs was closely associated with tumor progression and poorer survival in gastric cancer patients [51]. CAFs demonstrate a high level of basal secretory autophagy in head and neck squamous cell carcinoma. Secretory autophagy is involved in the export of cellular inflammatory mediators such as IL-6 and CXCL8. Combination therapy using autophagy inhibition with cisplatin significantly reduced tumor volume [52]. 

Stromal cells such as CAFs and MSCs enhance the triple-negative subtype of breast cancer metastasis-related phenotypes, including angiogenesis, migratory, and invasive properties, by releasing inflammatory chemokines such as CCL2 and CXCL8 [53]. Notch 1 activation is required for the induction of CXCL8 in tumor–stroma interactions and consequently for prometastatic activities [54]. Androgen receptor signaling in CAFs affects prostate cancer cell migration mediated by CXCL8 and CCL2 [55]. CAFs express CXCR2 and respond to paracrine signals of pancreatic cancer cells by upregulating CXCR2 ligands such as CXCL1, CXCL7, and CXCL8. CXCR2 knockout in a pancreatic ductal adenocarcinoma syngeneic mouse model suppressed angiogenesis and induced an antitumor response, but increased CAF activation, fibrosis, and metastasis in a mutation-dependent manner [56]. 

The CAF response to chemotherapy is highly variable and may impact on cancer therapy outcome [57]. Traditional maximum-tolerated dose chemotherapy induces CAF activation and results in the expression and secretion of ELR motif-positive chemokines such as CXCL6 and CXCL8 [58]. These chemokines signal though CXCR2 on cancer cells to transform into tumor-initiating cells, thus promoting aggression and treatment resistance. While the same overall dose administered as a low-dose metronomic chemotherapy largely prevented CAF activation and enhanced treatment response, it improved survival in mice [58]. CAF-secreted IL-6 and CXCL8 induce Bromodomain-containing protein 4 (BRD4) protein expression and lead to chromatin remodeling and Bromodomain and Extraterminal (BET) inhibitor resistance in colorectal cancer (CRC). Inhibition of IL-6/CXCL8-JAK2 signaling sensitized BET inhibitors in a CRC mouse xenograft model [59]. Simultaneous blocking of IL-6 and CXCL8 can inhibit CAF-induced human melanoma cell invasiveness using neutralizing antibodies in a 3D spheroid invasion assay [60]. Senescent human fibroblasts can secrete IL-6 and CXCL8 to promote cancer cell invasion and metastasis [61]. Senescent CAFs are a pathologically relevant fibroblast population that secrete excess CXCL8 to promote pancreatic cancer invasion [62]. A new subset of CD10^+^GPR77^+^CAFs induce cancer stem cells’ (CSCs) enrichment and chemoresistance by secreting IL-6 and CXCL8 in cancer. CD10^+^GPR77^+^CAFs constitute a supporting niche for CSCs, and its high expression correlates with poor survival in breast and lung cancer patients [63].

### 3.2. Microbiome in the TME

Commensal microbiota not only affect tumorigenesis, progression, and host metabolism but also shape the TME and regulate the efficiency of cancer therapy [64]. Bacteria induce an inflammatory response and stimulate epithelial cells to produce CXCL8, which can promote neutrophil recruitment. The mechanism of gut microbiome functions in cancer includes manipulating the gut epithelial barrier, modulating lymphoid organ activities, and regulating tumor immunity [65] and the TME via communication with various immune cells. The gut microbiota can signal to the central nervous system by producing hormones or immune-mediated signals through the gut–brain axis to affect tumor progression [66]. In a study of hundreds of hospitalized patients with cancer receiving allogeneic hematopoietic cell transplantation, the gut microbiota composition was closely associated with immune cell dynamics in humans after analyzing the daily changes of circulating neutrophil, lymphocyte, and monocyte counts [67].

#### 3.2.1. Dysbiosis and FMT

Dysbiosis is the imbalance of human microbiota composition mainly caused by drug exposure (antibiotics [68] and non-antibiotics [69]), alcohol overdose [70], or inappropriate diet [71]. Dysbiosis of the salivary microbiome contributes to the progression of oral squamous cell carcinoma (OSCC) by increasing inflammatory cytokines such as IL-6, CXCL8, TNF-α [72]. Dysbiosis increased the severity of amebic colitis through decreasing CXCR2 expression and neutrophil recruitment to the gut [73]. Antibiotic-induced dysbiosis enhances distal tumor progression through ICAM-1-mediated suppression of effector CD8+ T-cell trafficking into the tumor [74]. A high-fat diet promoted carcinogenesis by altering the esophageal microenvironment and gut microbiome via CXCL8/CXCL1/KC chemokines [75]. Chronic gastrointestinal diseases such as inflammatory bowel diseases (IBD), primary sclerosing cholangitis (PSC), cirrhosis, and tumorigenesis are promoted during dysbiosis conditions, which are associated with intestinal barrier dysfunction. Gut dysbiosis and intestinal barrier dysfunction are commonly observed in patients with PSC. A commensal Gram-negative gut microbiome can control the accumulation of CXCR2^+^ PMN-MDSC through the lipopolysaccharide (LPS)/TLR4/CXCL1 mechanism and promote cholangiocarcinoma [76]. 

Gut microbiota dysbiosis can be modulated through probiotics [77], fecal microbiota transplant (FMT) [78], engineered exogenous microbes, and bacteriophage therapy [79]. Preclinical and clinical evidence supports gut bacteria in modulating the efficacy of immunotherapy in cancers. Probiotic intervention in CRC patients altered the microbiota composition and increased microbial diversity [80]. Oral pretreatment with probiotic Bifidobacterium alleviates intestinal inflammation and colitis-associated cancer through CXCR2 signaling [81]. FMT promoted tumor response to anti-PD-1 immunotherapy in patients with refractory metastatic melanoma in a phase I clinical trial (NCT03353402). This study demonstrated an increased lamina propria infiltration of CD68^+^ cells. These increased antigen-presenting cells such as DCs in the gut could migrate into the TME through the lymphatic system and result in trafficking T cells into tumors [82]. Another single-arm phase I clinical trial (NCT03341143) enrolled sixteen patients with refractory metastatic melanoma, and demonstrated that the combination of FMT with anti-PD-1 therapy successfully induced persistent perturbation of the recipients’ gut microbiome and reprogrammed the TME to overcome primary resistance to anti-PD-1. Higher percentages of CD56^+^CD8^+^ T cells and mucosal-associated invariant T cells and lower percentages of CXCL8-producing myeloid cells were found both in PMBC and tumor samples after treatment [83]. LPS can stimulate the intestinal epithelial cells and activate proinflammatory signaling including phosphorylation of IRAK and MAP kinases and increased CXCL8 secretion [84].

#### 3.2.2. Intratumor Microbiome

The intratumor microbiome is also an integral part of the TME and may play a critical role in shaping the TME, affecting tumorigenesis, progression, and tumor immunity in the TME and the response to immunotherapy [85]. Lung cancer is closely associated with local dysbiosis, chronic inflammation, and pulmonary infections. Commensal microbiota induce proliferation and activation of IL-17-producing γδT cells to promote neutrophil infiltration and tumor development [86]. After a comparison of the inflammatory cytokine concentrations of saliva from head and neck squamous cell patients, CXCL8 and IL-1 β were significantly high and positively correlated with the abundance of oral microbe *C. albicans* [87]. Irrespective of the gut microbe or intratumor bacteria, gaining a better understanding of their role in the TME and response to immune therapy may pave the way for novel treatment options for cancer patients.

### 3.3. Crosstalk of the CXCL8-CXCR1/2 Axis and Immune Cells in the TME

Both CAF and the microbiome work indirectly to affect immunotherapy efficacy. Cytotoxic T cells can directly kill the cancer cells that need the infiltration, activation, proliferation, and spatial distribution of T cells within the TME. However, tumors have evolved many methods of escaping immune attack through recruiting neutrophils, MDSCs, or TAMs to the TME by cytokines or chemokines. The CXCL8-CXCR1/2 axis has been shown to be tightly connected with the neutrophil or MDSC recruiting, and CXCL8 can directly or indirectly affect the function of almost all immune cell types, thereby influencing tumor development. DCs produce CXCL8 and express the CXCL8 receptors CXCR1 and CXCR2. Human malignant cells can also produce CXCL8 to attract and retain DC to the tumor tissue that eventually decreases antitumor immunity [88]. 

#### 3.3.1. Neutrophils, MDSCs, and NETs

Neutrophils have many functions, such as phagocytosis, degranulation, reactive oxygen species (ROS) production, and extrusion of neutrophil extracellular traps (NETs) [89]. MDSCs contribute to tumor immune evasion through T-cell suppression, oxidative stress, and nutrient depletion. MDSCs are immature myeloid cells including monocytic (M-MDSCs) and granulocytic (GrMDSCs) subsets. Interactions between CXCR2 and CXCR2 ligands attracted MDSC traffic to the TME and enhanced rhabdomyosarcoma progression in a murine model. CXCR2 inhibition prevented MDSC trafficking to the TME and improved the efficacy of anti-PD-1 therapy [90]. TNBC cell autocrine CXCL8 blocked with anti-CXCL8 monoclonal antibody HuMax-IL8 reverted mesenchymalization and decreased recruitment of PMN-MDSCS in the TME, especially when combined with chemotherapy and immune-based therapy [91]. Blocking CXCR1 and CXCR2 with small molecule inhibitor SX-682 significantly abrogated CXCR2^+^PMN-MDSC tumor accumulation and enhanced the efficacy of both the PD-axis immune checkpoint blockade and adoptive cell transfer of engineered T cells [92]. SX-682 can also improve the NK cell-based immunotherapy efficacy through inhibiting CXCR1/2-related PMN-MDSC trafficking in head and neck cancer models [93]. Combining SX-682 with a bifunctional anti-PD-L1 and transforming growth factor (TGF- β) agent bintrafusp alfa (M7824) decreased mesenchymal markers and improved antitumor activity [94]. The mechanism included inhibiting MDSC migration, enhancing CD4^+^/CD8^+^ T-cell infiltration, and remodeling the TME. MDSC accumulation is associated with intratumoral expression of IL1β, IL8, CXCL5, and Mip-1α, and MDSCs levels were increased in the peripheral blood and tumor of patients with renal cell carcinoma. Increasing PMN-MDSC levels in the peripheral blood correlate with a higher tumor grade. A CXCR2 antagonist decreased PMN-MDSC recruitment and enhanced CD4^+^ and CD8^+^ T-cell infiltration and the efficacy of anti-PD-1 therapy in mice bearing renal tumors [95]. 

NETs are extracellular web-like structures composed of granule proteins and DNA fibers. NETs can constrain and kill invasive pathogens and prevent bacterial dissemination. A recent study demonstrated that NETs may be involved in pathogenesis and tumor progression [96]. Tumor-produced CXCL8 attracts MDSC to the TME and elicits granulocytic MDSC to extrude NETs for nesting tumor cells [97]. A further study demonstrated that CXCL8 induced tumor-associated neutrophil and granulocytic MDSC extrusion of NETs, which shield tumor cells from contact with CD8^+^ T cells and limit the anti-PD-1 immune response to cancer [98]. Conclusive evidence showed that multiple human cancer types contain NETs, which had a positive association with CXCL8 and reduced CD8^+^ T-cell infiltration [99]. Tumorous CXCL8 mediates increased NETs formation in the TME, promoting colorectal cancer liver metastasis [100]. CXCL8 knockdown inhibits tumor growth in colorectal liver metastasis [101]. Cancer cells can secrete exosomes to regulate the TME. Exosomes transfer mutant KRAS to neutrophils and increase CXCL8 production and NET formation to promote CRC proliferation [102]. Nets are produced by tumor-infiltrating neutrophils involved in glioblastoma cell proliferation and invasion through stimulation of the nuclear factor kappa B (NF-κB) signaling pathway, which promoted CXCL8 secretion in glioblastomas [103]. The CXCL8/CXCR2 axis mediates the formation of NETs, which promotes proliferation and migration through TLR9 signaling in diffuse large B-cell lymphoma [104]. 

CXCR2 signaling is upregulated much more in neutrophil/MDSCs than in human pancreatic cancer cells and is associated with poor prognosis in patients. Inhibition of CXCR2 signaling reduces tumor metastasis, enhances sensitivity to anti-PD1 immunotherapy, and prolongs survival in mice [105]. IL-6/CXCL8 induces MDSC arginase I production to suppress CD8^+^ T-cell activity through the PI3K-Akt signaling pathway in gastric cancer [106]. Receptor-interacting protein kinase 3 (RIP3) knockdown promotes tumor progression and immune escape through driving MDSC recruitment by CXCR2 and decreasing interferon (IFN)-γ^+^ CD8^+^ T cells in hepatocellular carcinoma (HCC) [107]. Tumor cells induce MDSC recruitment in the bladder cancer TME through CXCL2/MIF-CXCR2 signaling, which is correlated with poor prognosis [108]. CXCL8 contributes to the recruitment of tumor-associated neutrophils that suppress CD8^+^ T-cell activity, partly depending upon Notch signaling in ovarian cancer [109]. The expression of CXCR2 is higher in tumor stroma than tumor cells in human lung cancer and correlates with poor prognosis. Blockade of CXCR2 decreasse neutrophil infiltration in the TME and enhances antitumor T-cell activity and the antitherapeutic effects of cisplatin in lung cancer [110].

#### 3.3.2. Macrophages and TAMs

TAMs constitute an important component of the TME, and can be divided into M1 macrophages and M2 macrophages. M2 macrophages can improve tumor progression and facilitate tumor immune escape. Overexpressed CXCL8 in pancreatic cancer mediates CXCR2^+^CD68^+^ macrophage trafficking to the TME and contributes to cancer progression and PD-1 blockade resistance. A mechanism study found that IFN-γ suppresses the expression of CXCL8 in pancreatic cancer and inhibits TAMs migration to enhance anti-PD1 efficacy [111]. TAMs secrete CXCL8 to promote pancreatic cancer cell migration and invasion through the signal transducer and activator of transcription 3 (STAT3) pathway [112]. Neurotensin induces the secretion of CXCL8 in HCC cells. Dysfunctional activation of the neurotensin/CXCL8 pathway is associated with more EMT and worse prognosis in HCC patients [113]. HCC-derived CXCL8 enhances tumor invasion and migration by promoting M2-type TAM accumulation and EMT [114]. Macrophage-derived CXCL8 induces EMT by activating the JAK2/STAT3/Snail pathway in HCC cells [115]. CXCL8 secreted from M2 macrophages promotes prostate tumorigenesis and proliferation of prostate cancer cells in vitro [116]. TAM-derived CXCL8 enhances thyroid papillary cancer invasion and metastasis [117]. 

CXCL8 promotes HCC invasion and metastasis by upregulating FoxC1 expression and enhances macrophage infiltration in mice [118]. The CXCL8/STAT3 pathway plays a key role in M2 macrophage polarization and stemness of ovarian cancer stem-like cells [119]. A further study demonstrated that genistein can suppress M2 polarization of macrophages and stemness of ovarian cancer through CXCL8/STAT3 signaling [120]. CXCL8 derived from Tregs is higher than TAMs and MDSCs in malignant pleural effusion. CXCL8 further induces TGF-β upregulation in M2 macrophages and mediates CCL22 production from TAMs, which promots an immunosuppressive TME in lung cancer [121]. CXCL8 and PAI-1 secreted from oral squamous cell carcinoma promote the differentiation of monocytes to TAMs [122]. Tumor-derived IL-6/8 impairs the function of NK cells through STAT3 signaling and enhances malignancy in primary esophageal squamous cell carcinoma [123].

#### 3.3.3. EMT and Stemness

CXCL8 exerts protumor effects on the TME, such as activating EMT and enhancing stemness and therapy resistance. EMT is known to promote tumor cell motility, invasiveness, and metastasis of solid cancers. CXCL8 is an important regulator of the TME by promoting EMT and stemness in cancer cells [124]. CXCL8 secreted by tumor cells could enhance tumor progression by EMT in breast cancer cells [125]. 

The stemness of the breast cancer cell line MCF-7 was increased after CXCL8 stimulation in a tumorsphere-formation assay [126]. Overexpresson of CXCL8 promotes cell proliferation and migration through EMT, PI3K-Akt signaling, and E-cadherin downregulation in triple-negative breast cancer [127]. CXCL8 enhances the stemness properties of the self-renewal capability, expression of stemness-related genes, and tumorigenicity in small-cell lung cancer sphere-forming cells [128]. IL-6 and CXCL8 promote EMT and cell invasion through STAT3 signaling in oral cancer [129]. Snail is a key transcriptional repressor of E-cadherin that induces EMT and promotes tumor progression. An CXCR2 antagonist suppresses MDSC infiltration and inhibits ovarian cancer progression through the Snail/NF-kB axis [130]. Sulconazole inhibits breast cancer cell proliferation and cancer stem cell formation via the NF-κB/CXCL8 signaling pathway [131]. Therapeutic stress increases CXCL8 and CXCR2 expression, enhancing glioblastoma tumorigenicity, growth, and therapy resistance by inducing glioma-initiating cell formation [132]. CXCL8 enhances cancer stem cell-like properties such as sphere formation, glucose uptake, SOX2, and GLUT3 expression that promote xenograft tumor growth in lung and colon cancer cells [133]. CXCL8 enhances tumor proliferation and metastasis through the interaction between human osteosarcoma and mesenchymal stem cells in the TME [134]. Mesenchymal stem cell-derived CXCL8 promotes osteosarcoma cell anoikis resistance and pulmonary metastasis through CXCR1/Akt signaling [135]. CXCR1 gene knockdown improves the sensitivity of human osteosarcoma to cisplatin both in vivo and in vitro [136]. 

#### 3.3.4. Angiogenesis

Angiogenesis plays a key role in tumor progression and metastasis. CXCL8 enhances angiogenesis and tumor growth through Ras/MAPK/PI3K activation [137]. CXCL8 inhibition led to an impairment of tumor vascularization and tumor death. NF-κB enhances the secretion of CXCL8 and VEGF to induce angiogenesis in pancreatic cancer cell lines [138]. CXCR2 significantly promotes angiogenesis, proliferation, and invasion of pancreatic cancer [139]. Pancreatic cancer cell increases CXCL8 secretion to enhance angiogenesis and acquisition of gemcitabine resistance [140]. CXCR1 promotes proliferation, migration, invasion, and metastasis in gastric cancer in vitro and in vivo [141]. CXCR1 depletion reduces angiogenic potential and decreases tumor growth in androgen-independent prostate cancer [142]. CXCL8 contributes to angiogenesis and tumor progression in glioma through the AP-1/NF-kB axis [143]. CXCL8 stimulates the proliferation and migration of vascular endohthelial cells to promote angiogenesis and tumor growth [144]. Activated endothelial cells secrete CXCL8 to promote proliferation and chemoresistance of acute myeloid leukemia [145]. 

CXCL8 enhances proliferation and migration through metadherin in gastric cancer in a xenografted mice model [146]. Overexpressed CXCL8 promotes intergrin β3 upregulation and cell migration through the PI3K/Akt pathway in estrogen receptor-negative breast cancer [147] and HCC [148]. CXCL8 enhances cell migration through increasing integrin αvβ6 expression in colorectal cancer [149]. Pyruvate dehydrogenase kinase 1 (PDK1) promotes ovarian cancer cell angiogenesis, invasion, and metastasis through α5β1 integrin and JNK/CXCL8 signaling [150]. CXCL8 and CXCL2 enhance angiogenesis through CXCR2 signaling on endothelial cells in glioblastoma [151]. Probibitin 1 decreases CXCL8 expression to inhibit tumorigenesis through NF-κB/AP-1 signaling in human liver cancers [152]. Sunitinib inhibits tumor angiogenesis through targeting VEGF or PDGF receptors in renal cell carcinoma. CXCL8 is an important contributor to sunitinib resistance, and CXCL8 blocking resensitizes renal cell carcinoma to sunitinib treatment [153]. 

#### 3.3.5. CXCL8 with Various Signaling Pathways

Instead of the above mechanism, the CXCL8-CXCR1/2 axis have many other roles in cancer. Activated pancreatic stellate cells enhance pancreatic cancer fibrosis and progression through paracrine CXCL8 [154]. Activated hepatic stellate cells secrete CXCL8 to induce the proliferation of breast cancer cells and stimulate dormant cancer cells to grow in a 3D liver microphysiological system [155]. CXCL8 released from pancreatic cancer induces muscle atrophy to cause involuntary body weight loss or cachexia in patients [156]. Hypoxia-treated CRC tumors show increased CXCL8 expression and promote the migration, invasion, and metastasis of normoxic CRC cells [157]. Blocking the JNK/CXCL8 pathway enhances radiation-induced necroptosis and radiotherapy efficacy in colorectal cancer [158]. IL-6 and CXCL8 together promote cell density-dependent migration and the inhibition of the synergistic IL-6/CXCL8 signaling pathway reduces metastatic burden in mice [159]. CXCL8 mediates the effect of augmented FOXA1 on cell growth, invasion, and endocrine resistance in estrogen receptor-positive breast cancer [160]. Ataxia-telangiectasia mutated (ATM) protein kinase regulates CXCL8 to enhance cell migration, invasion, and metastasis in breast cancer [161]. Necrotic cells induce CXCL8 expression through AP-1/NF-κB activation that promote cell migration and invasion in glioblastoma [162]. PENT plays a key role in STAT3 activation and CXCL8 secretion in tumors. CXCL8 promotes tumor progression through the STAT3 signaling pathway in head and neck squamous cell carcinoma [163].

## 4. Therapeutic Targeting of the CXCL8-CXCR1/2 Axis in Cancer 

**Figure 3:**
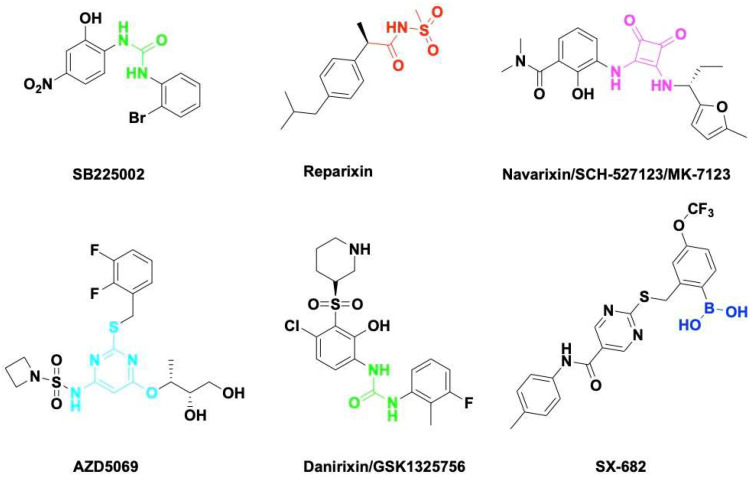
Small molecule antagonists targeting CXCR1/2. Blocking CXCR1 or CXCR2 impairs immune suppressive cell recruitment and angiogenesis to enhance cancer therapy efficacy.

Chemokine CXCL8 activates both CXCR1 and CXCR2. All of them have been shown to be upregulated in acute or chronic inflammatory diseases including cancer. CXCR1 and CXCR2 constitute the primary mechanism for the recruitment of neutrophils and MDSCs, which could enhance tumor progression and suppress immune therapy efficacy. Blocking the CXCL8-CXCR1/2 axis with small molecules (Figure 3) or antibodies should be a promising therapeutic strategy to overcome immune suppression in the TME. 

SB225002 was first reported as a small molecule CXCL8 inhibitor binding to CXCR2. SB225002 selectively blocked CXCL8-induced neutrophil chemotaxis and margination in rabbits [164]. Blocking CXCR2 with SB225002 has proved to inhibit tumor progression in breast cancer [165], ovarian cancer [166], acute myeloid leukemia [167], and nasopharyngeal carcinoma [168]. Reparixin is a non-competitive allosteric inhibitor of CXCR1 and CXCR2 that could inhibit polymorphonuclear cell recruitment in vivo. The activity of Reparixin on CXCR1 was 100-fold higher than on CXCR2 [169]. CXCR1 has been identified as a druggable target for breast cancer stem cells [170]. 

Navarixin is an orally selective, CXCR1 and CXCR2 receptor antagonist that impairs neutrophil recruitment in rodents and monkeys [171]. Navarixin decreased tumor cell proliferation and angiogenesis in a melanoma mouse model [172]. A phase II clinical trial (NCT03473925) assessed the efficacy and safty of navarixin in combination with anti-PD1 pembrolizumab in NSCLC, Castration-Resistant Prostate Cancer and Colorectal Cancer (Table 1). AZD5069 is a reversible CXCR2 antagonist that inhibits CXCL8 binding and neutrophil chemotaxis [173]. AZD5069 treatment inhibits TAM infiltration and vessel formation, increases CD4^+^/CD8^+^ T-cell infiltration, and suppresses tumor growth in advanced prostate cancer [174]. Combination therapy of AZD5069 with anti-PD1 Durvalumab (MEDI4736) was studied in patients with recurrent and/or metastatic squamous cell carcinoma of the head and neck (NCT02499328) and metastatic pancreatic ductal adenocarcinoma (NCT02583477). Danirixin is also a reversible and selective antagonist of CXCR2 that may be of benefit in diseases of excess neutrophilia [175]. The clinical trial of danirixin in healthy, elderly human volunteers demonstrated that it was safe and tolerable, and no serious adverse events were reported [176,177]. A recent study showed that danirixin suppressed breast cancer migration, invasion, and metastasis mediated by CXCL8 and TAMs [178]. 

SX-682 is a novel CXCR1/2 chemokine receptor boronic acid antagonist with potential anticancer activities. Orally bioavailable SX-682 enhanced NK cell activation and therapeutic efficacy by inhibiting MDSC accumulation in the TME [93]. A combination of SX-682 with anti-PD1 caused a reduction in tumor burden b7 increasing CD8+ T-cell infiltration and decreasing neutrophil accumulation in non-small cell lung cancer [179]. Treatment of tumor-bearing mice with SX682 reduced intertumoral MDSCs, increased CD8+ T-cell recruitment, and inhibited tumor growth in melanoma and breast cancer [180]. Several clinical trials were designed to assess the efficacy of SX-682 in combination with anti-PD1 Nivolumab or Pembrolizumab in metastatic melanoma, colorectal carcinoma, and pancreatic cancer (Table 1). Fully humanized neutralizing antibody ABX-IL8 inhibits angiogenesis, tumor growth, and metastasis in melanoma [181] and bladder cancer [182] through downregulation of MMP-2. HuMax-IL8(BMS-986253) is another fully human anti-CXCL8 monoclonal antibody. A phase I clinical trial of HuMax-IL8 (NCT02536469) showed no objective tumor responses, but it is safe and well tolerated [183]. Phase II clinical trials of HuMax-IL8 plus anti-PD1 Nivolumab are ongoing in patients with advanced solid tumors (Table 1). 

Chimeric antigen receptor (CAR) T-cell therapy has shown clinical efficacy for hematological malignancies but still remains a challenge for solid tumors [184]. The major obstacles of CAR-T therapy in solid tumors are tumor heterogeneity, an immunosuppressive TME, and T-cell trafficking/infiltrating to the tumor. CXCR1/2-modified CAR-T cells enhance T-cell trafficking, persistence of T cells in the tumor, long-lasting immunologic memory, and therapy efficacy in aggressive solid tumors such as glioblastoma, ovarian, and pancreatic cancer [185]. 

## 5. CXCL8 as a Prognosis Biomarker in Cancer Therapy

Tumor burden (or tumor size) predicts response to immunotherapy in patients with cancer. A large TME and a small TME are characterized by different cell populations and responses to specific interventions [186]. Radiologic imaging such as computed tomographhy (CT) is the most commonly used technology for tumor burden monitoring even though it has limitations for the evaluation of the response to immunotherapy. Functional imaging techniques [187] such as positron emission tomography (PET) or electron paramagnetic resonance (EPR) can sense the TME hypoxia and visualize tissue redox status non-invasively, which assists image-guided diagnoses and efficacy evaluations of cancer therapy [188]. CXCL8 serum concentrations can accurately reflect the tumor burden of patients following antitumor therapy and have prognostic significance [189].

CXCL8 could be a serum biomarker by which to predict clinical benefit from immune checkpoint blockade in melanoma and NSCLC patients. Serum CXCL8 levels significantly decreased in responding patients treated with anti-PD1 nivolumab or pembrolizumab, which were associated with longer overall survival [190]. In gemcitabine-refractory patients with pancreatic cancer, plasma CXCL8 is a useful circulating biomarker for predicting resistance to nanoliposomal irinotecan therapy [191]. Baseline IL-6/CXCL8 can predict objective response and overall survival in patients with advanced HCC treated with sorafenib [192]. 

## 6. Conclusions and Prospective

As a key driver of immune suppression, CXCL8-CXCR1/2 axis blocking is a promising way to make the TME much more conductive to a therapeutic response. The present studies suggest that the CXCL8-CXCR1/2 axis has a key role in determining the types and quantity of immune-suppressive cells infiltrating cancers. The CAFs and microbiome affect the CXCL8 levels to modulate the immune homeostasis in the TME. An easily measurable prognostic biomarker is valuable for patients treated with immunotherapy since not all patients respond to immunotherapy. A large-scale retrospective analysis demonstrated that serum CXCL8 levels are associated with increased monocyte and neutrophil infiltration as well as worse prognosis in advanced cancer patients with immune checkpoint inhibitors [193]. Similar results were achieved in a large randomized study in which a high circulating CXCL8 level correlated with reduced clinical benefit of PD-L1 blockade in patients with metastatic urothelial carcinoma and metastatic renal cell carcinoma [194]. These studies suggest that plasma CXCL8 may be a prognostic biomarker in solid tumors and can be used to minimize toxicity and maximize clinical benefit. The combination of blocking the CXCL8-CXCR1/2 axis with other types of immunotherapy is a promising strategy by which to extend the durability of clinical responses in patients with cancer. Nevertheless, more clinical trials are needed to validate the CXCL8-CXCR1/2 axis in both therapeutic and prognostic roles in cancer immunotherapy.

## Figures and Tables

**Table 1:** Selected clinical trials targeted in CXCL8-CXCR1/2 axis.

Drug (Manufacturer)	Target	Therapeutic Combinations	Cancer Type	Phase	Clinical Trials	Recruitment Status
SX-682 (Syntrix Biosystems, Inc.)	CXCR1/2	Pembrolizumab (anti-PD-1)	Metastatic Melanoma	Phase I	NCT03161431	Recruiting
		Nivolumab (anti-PD-1)	Metastatic Colorectal Carcinoma	Phase Ib/II	NCT04599140	Recruiting
		Nivolumab (anti-PD-1)	Pancreatic Cancer	Phase I	NCT04477343	Recruiting
		Bintrafusp alfa (anti-PD-L1/TGF-β) CV301 (cancer vaccine)	Advanced Solid Tumors	Phase I	NCT04574583	Active, not recruiting
AZD5069 (AstraZeneca)	CXCR2	Durvalumab (anti-PD-L1)	Advanced Solid Tumors	Phase Ib/II	NCT02499328	Active, not recruiting
		Durvalumab (anti-PD-L1)	Metastatic Pancreatic Ductal Adenocarcinoma	Phase II	NCT02583477	Completed
Navarixin (Merck Sharp & Dohme Corp.)	CXCR1/2	Pembrolizumab (anti-PD-1)	Advanced Solid Tumors	Phase II	NCT03473925	Completed
HuMax-IL8 (Bristol-Myers Squiibb)	CXCL8	Nivolumab (anti-PD-1)	Head and Neck Squamous Cell Carcinoma	Phase II	NCT04848116	Recruiting
		Nivolumab (anti-PD-1)	Prostate Cancer	Phase Ib/II	NCT03689699	Recruiting
		Nivolumab (anti-PD-1)	Pancreatic Cancer	Phase II	NCT02451982	Recruiting
		Nivolumab (anti-PD-1)	Hepatocellular Carcinoma	Phase II	NCT04050462	Recruiting
		Nivolumab (anti-PD-1)	Advanced Cancers	Phase I/II	NCT03400332	Recruiting
		Nivolumab (anti-PD-1)	Non-small Cell Lung Cancer	Phase II	NCT04123379	Recruiting
